# Capsule Endoscopy in Patients with Cardiac Pacemakers, Implantable Cardioverter Defibrillators, and Left Heart Devices: A Review of the Current Literature

**DOI:** 10.1155/2011/376053

**Published:** 2011-04-27

**Authors:** Dirk Bandorski, Martin Keuchel, Martin Brück, Reinhard Hoeltgen, Marcus Wieczorek, Ralf Jakobs

**Affiliations:** ^1^Universitätsklinikum Gießen, Medizinische Klinik 2, Klinikstraße 36, 35392 Gießen, Germany; ^2^Bethesda Krankenhaus Bergedorf, Glindersweg 80, 21029 Hamburg, Germany; ^3^Klinikum Wetzlar, Medizinische Klinik 1, Forsthausstraße 1, 35578 Wetzlar, Germany; ^4^Herzzentrum Duisburg, Medizinische Klinik III, Gerrickstraße 21, 47137 Duisburg, Germany; ^5^Klinikum Ludwigshafen, Medizinische Klinik C, Bremserstrasse 79, 67063 Ludwigshafen, Germany

## Abstract

*Background and Study Aims*. Capsule endoscopy is an established tool for investigation of the small intestine. Because of limited clinical experience in patients with cardiac devices, the Food and Drug Administration and the manufacturer recommended not to use capsule endoscopy in these patients. The vast majority of investigations did not reveal any interference between capsule endoscopy and cardiac devices. *Methods*. Studies investigating interference between CE and cardiac devices were analysed. For the review we considered studies published in English or German and indexed in Medline, as well as highly relevant abstracts. *Results*. In vitro and in vivo studies mainly revealed no interference between capsule endoscopy and cardiac devices. Technical data of capsule endoscopy (Given Imaging) reveal that interference with cardiac pacemakers and implantable cardioverter defibrillator is impossible. Telemetry can interfere with CE video. *Conclusion*. The clinical use of capsule endoscopy (Given Imaging) is unproblematic in patients with cardiac pacemakers.

## 1. Introduction

Capsule endoscopy (CE) is an established tool for investigation of the small intestine. Because of limited clinical experience of CE in patients with cardiac pacemaker (PM)/implantable cardioverter-defibrillators (ICDs), the Food and Drug Administration, as well the manufacturers Given Imaging and Olympus, recommended not using CE in these patients. The vast majority of published investigations did not reveal any interference between small bowel capsule endoscopy (Given Imaging Ltd., Yogneam, Israel; Olympus Medical Systems Corp., Tokyo, Japan), Pill Cam Colon (Given Imaging Ltd., Yogneam, Israel) on the one hand, and cardiac pacemakers and ICD on the other hand. For this paper we considered studies published in German or English and indexed in Medline and current abstracts of high relevance. 

## 2. Capsule Endoscopy in Patients with Cardiac Pacemakers

Interference between capsule endoscopy and cardiac pacemaker (PM) had been evaluated in several studies (Table 1). 

The study of Dirks et al. investigated 5 patients with a cardiac pacemaker who underwent CE [1]. In two patients with an abdominal pacemaker, the authors screened the patients for interference by placing the capsule on the abdominal wall of the patient in proximity to the abdominal PM. The PM was set in VVI mode for a back-up rate of 30 bpm, and analysis for normal functioning was performed with the magnetic programmer. All patients included in the study were closely monitored for the duration of the CE in their outpatient investigation unit. No adverse events were documented, and the quality of the video was without any interference. 

Our in vitro study investigated 21 PMs from 7 brands [2]. The pacemakers used for the investigation came from a collective gathered over 4 years. They had mainly been removed from deceased patients before cremation. All PMs were tested for normal functioning. To electrically simulate the situation of a pacemaker inside a patient, the PM and its lead were positioned in a saline solution with a resistivity corresponding to that of the lower frequency range of muscle tissue, analogously to a study of Irnich et al. in which the interference behaviour of mobile phones with respect to PM was investigated [3]. Briefly, a 0,9 g/L saline solution has been used. Its conductivity of 170 mS/m (5,9 Ωm correspondingly) was chosen to simulate the low frequency behaviour of tissue. The PM pulse was registrated with pin jacks which were placed fairly low down at the bottom of the tank. The jacks also allowed the input of an inhibiting signal. At the beginning of the experiment, the sensitivity settings were left as they had been in the patient. The devices were then programmed to the maximum sensitivity. First the pacemakers were checked while operating with an unsynchronized impulse and a minimum distance between PM and capsule which was placed, for 1 minute each time, in different positions and directions relative to the PM and finally on the case of the PM. Then the pacemaker was inhibited corresponding to the CENELEC standard test signal. Measurements were then repeated with the capsule in the different positions and orientations for 1 minute each time. The function of the capsule was registrated with the aid of receiving electrodes and the data passed to a recording device. None of the PM tested, in either the asynchronous or synchronous mode, showed interference from a capsule. Functioning of the capsule or the quality of the recorded signal was not influenced by PM. 

Previously we performed an in vitro study with one pacemaker (Philos DR, Biotronik, Berlin, Germany) and CE (Given Imaging) which was placed in a cleaned porcine gut [4]. The gut was inserted in a horizontal direction into a plastic bag filled with glucose solution. With the eight sensors, the transmitting signals of CE were sent to a receiving unit. The PM (DDD-Mode) was placed in proximity of the gut/CE opposite to the electrodes. The function of the PM was observed permanently. Finally an electrode with the cardiac pacemaker in VVI-Mode (uni- and bipolar stimulation), was placed in the glucose solution near the gut. An ECG was recorded for about 10 minutes. There were no interferences between the CE and the PM in spite of the close proximity of CE and PM. 

The study of Payeras et al. had two phases, an in vitro and an in vivo one. The objective of the in vitro study was to investigate interference between CE and PM [5]. A PM (Kappa KD 701, Medtronic, Minneapolis, MN, USA) was connected to an interference detector which displays a graph of PM rhythm. Possible interference between PM and CE was tested for 1 minute with a TestCap (Given Imaging, Yogneam, Israel), a device that transmits an indentical signal as the endoscopic capsule (personal information of the manufacturer) but without optical properties. First possible interference between PM and CE was assessed in an air medium and then with the pacemaker placed into a vessel containing a solution, similar to the one used in our investigation. In each medium, the experiment was performed twice, first with the PM in a unipolar and then in a bipolar mode. The authors did not exactly describe the procedure of testing interference between CE and PM. In the in vivo part first a test capsule was placed close to the patient's chest and consecutively the patient swallowed CE. All patients were connected to a Holter monitor for the duration of CE. None of the PM was influenced by the TestCap or CE. No defects in the quality of the images occurred in any patient.

A prospective inquiry in the year 2004 evaluated the experience related to CE in patients with electrical implants (PM/ICD) in Germany [6]. A standardized questionnaire was sent to all centers in Germany performing CE. The questionaire covered the number of examined patients, indication for CE, quality of CE videos, type of cardiac monitoring during CE, check of the electric implants before and after CE, occurrence of arrhythmia, and complications. In 28 centers, 45 patients with a PM and 8 patients with an ICD were examined with CE. There were no relevant complications. In only two cases, supraventricular extrasystoles were recorded. In two patients with a PM, artifacts in the CE-video were seen. 

Dubner's study is the only one with interference between CE and PM. In this study, 100 patients with an implanted PM were included [7]. The testing was performed with a TestCap (technical properties identical to CE). Continuous electrocardiographic monitoring was performed during the study. The TestCap was placed above the pulse generator and then manually moved along the course of the leads to the atrial and ventricular tips. The test was performed 3 times, each time with the TestCap at a different distance: close (2 cm), around 10 cm from the skin surface and more than 10 cm from the surface for 10, 30, and 60 seconds. Patients with interference were reevaluated one week later. In this study interference was observed in 4 patients and was reproducible 1 week later. The interference occurred when the TestCap was localized within 10 cm of the skin surface close to the generator and the electrodes (manufactured by St. Jude Medical, St. Paul, MN, USA and Biotronik, Berlin, Germany) and caused the pacemaker to revert to noise-mode function (VOO- or DOO-Mode). The PM were programmed in bipolar sensing mode and there were no differences in the sensitivity of these devices compared with the PM for which testing was negative. The authors conclude that the observed interference between TestCap simulation and PMs in the setting of a “worst case senario” did not have clinical relevance. 

Guyomar et al. report about a patient with an abdominal VVI-PM connected to an unipolar epicardial electrode after an episode of endocarditis, which required the removal of all preexistent prosthetic material [8]. First CE was positioned over various abdominal sites near the pulse generator (programmed in VVI mode), while ECG was continuously recorded. No interference was recorded. Before swallowing the CE, the PM was programmed to VOO-Mode (70 bpm). The patient was monitored by a central station (telemetry?) for the duration of the endoscopy procedure. Neither interference between CE and PM nor PM dysfunction during the examination was observed. However, the CE-video revealed innumerable “blank periods” during the first half of the examination. The earliest study of Leighton et al. investigated 5 patients [9]. Before the CE procedure, an electrocardiogram was obtained and PM functions were checked. During CE, cardiac rhythm was monitored with a Holter ECG. After CE, PM function was checked for any disturbance. Atrial and ventricular extrasystoles were registered in 3 patients; one patient had a 3-beat-run of a nonsustained ventricular tachycardia. PM functions were not altered. There was no interference noted in the images. 

## 3. Capsule Endoscopy in Patients with Implantable Cardioverter Defibrillators

Several studies investigated patients with ICD who underwent CE (Table 2). Additionally, our in vitro study investigated interference between CE and ICD in vitro [10]. Two reactions of ICDs with respect to continuous interference are possible: the pacing pulses of the ICDs are inhibited by the interference voltages, or the ICDs, if inhibited by a test signal, operate in an asynchronous mode, the so-called interference mode. This reaction is comparable to that of pacemakers. In a pilot study, interference behaviour was tested in 47 ICDs with respect to continuous 50 Hz-voltages coupled directly into the device. They all (except the five Biotronik ICDs) came from a collection of ICDs gathered over 4 years, being explanted from deceased patients before cremation. The manufacturer Biotronik supported our investigation by providing five “Belos” ICDs. The 50 Hz-voltages were chosen because pacemakers and ICDs are most sensitive to this interference voltage [11]. All other interference voltage, thus, must have higher amplitudes than that measured with 50 Hz. The interference threshold was tested with a signal generator producing continuous 50 Hz voltages that was coarsely increased from below threshold to above threshold and then fine-tuned to exact threshold. A pilot study revealed that out of the 47 devices tested, 36 (77%) were inhibited (Group 1) whereas the remaining 11 (23%) elements were switched to asynchronous interference mode (Group 2). Differentiation of the ICDs concerning their interference behaviour in the two groups is necessary for the following interference investigation with endoscopy capsules. 

A total number of 47 ICDs were included in this investigation; two of them investigated in the pilot study could not be used because of connector problems. The test setting was created analogously to a study of Irnich et al. in which the interference behaviour of mobile phones with respect to pacemakers was investigated [3]. The ICDs and their lead(s) were positioned in a saline solution of 0.9% with a resistivity corresponding to that of the low frequency range of muscle tissue, to electrically simulate the situation of an ICD inside a patient. Quite low at the bottom of the tank, pin jacks were placed that were in contact with the solution. By these jacks, the ICD pulse was registered, and signals could be coupled in. The ICD pulses were registered by an oscilloscope. For ICDs of Group 1, interference, if present, should be characterized by inhibition of the pacing pulses. For ICDs of Group 2, a triangular test signal according to the European Standard EN 45502-2-1 (CEN/CENELEC) was coupled in with the aid of a function generator to inhibit the ICDs. Interference, if present, should be characterized by asynchronous pacing. The ICDs of both groups were checked with variable distances between ICD and capsules. The capsules were held by hand in different positions and directions to the ICD for 1 minute each. Finally, the capsules were placed directly on the case of the ICD for 1 minute. Attention was paid to possible interference effects. In addition, the capsules were positioned for 1 minute close to the tip, ring, and coil of the lead while the device was operating in asynchronous (Group 1) or synchronous (Group 2) mode. Function of capsules was recorded with the aid of receiving electrodes distributed on the body surface, and the data were passed to a recording device. During the whole investigation, the function of the ICDs was checked by registering the pacing pulses with an oscilloscope. None of the ICDs, operating in asynchronous or synchronous mode, showed interference caused by a capsule, or, in other words, no dysfunction of the ICDs of both groups was detected. This holds for all positions of the capsules with respect to the lead and the ICD devices investigated. No interactions between the capsules and the ICDs were seen. For the entire duration of our experiment, an undisturbed signal was sent by the capsules. In spite of the proximity between the capsules and the ICD, the function of the capsules or the quality of the recorded signal was not affected at all. 

Similar to their findings in patients with PM, Dubner et al. reported about interference between CE and ICD [12]. They performed a study with an in vitro and an in vivo part. For the in vitro part, they placed an ICD and its lead into a saline gel bath with a controlled temperature of 98°F. The probe associated with the TestCap (technical properties identical to CE, Given Imaging) was positioned close to the bath at specified distances and locations with respect to the ICDs. Tests were carried out at the nominal and most sensitive setting of the ICDs. The TestCap was located 1, 5, 10, and 15 centimeters from the ICD over three different positions (ring, coil, pulse-generator) during 10, 30, and 60 seconds. ICDs were checked before and after the test with specific programmers. To confirm the results and avoid false negative/positive results, tests were performed one week later. There was no inhibition of the brady therapy. Positive and reproducible interference (oversensing) was observed when the TestCap was placed over the ring and the coil but not over the pulse generator (Belos DR, Biotronik, Gerlin, Germany). Oversensing (triggering delivery of inappropriate therapy) could not be eliminated even at a distance of 30 cm from the ICD system. The in vivo part of the study investigated 6 ICD patients with devices that showed no interference during the in vitro study. Each ICD system was evaluated similar to the in vitro part. The ICDs were programmed to the manufacturers' nominal and most sensitive settings. The TestCap was located 1, 5, 10, and 15 centimeters to the skin each in three different positions (ring, coil, pulse-generator) during 10 and 30 seconds. The 60 second period was excluded by the authors for safety reasons. The ICDs tested in the in vivo part showed no interference. 

 Pelargonio et al. published a case report on the application of CE in a patient with an ICD (Medtronic, Minneapolis, MN, USA, GEM III 7275) [13]. Before and after CE, the ICD was interrogated. During CE, ECG was continuously monitored. No arrhythmia or other adverse cardiac events were recorded. The programmed parameters were not altered, and no interference was found between CE and the ICD. 

Our inquiry in the year 2004 (mentioned above) also evaluated the experience of CE in patients with electrical implants (PM/ICD) in Germany [6]. Eight patients with an ICD underwent CE without evidence for interference between ICD and CE. 

The first case series on patients with an ICD who underwent CE was published by Leighton et al. [14]. Five patients with ICDs were studied. Before CE (Given Imaging), all patients had a baseline ECG and an ICD interrogation. Thereafter, CE was performed. During CE the patients were monitored by telemetry during CE. A postprocedure ICD interrogation was carried out. In all patients, normal ICD functions and programmed parameters were not altered. Hemodynamically significant arrhythmias were not observed in any of the patients. The CE images were reviewed. There were no technical difficulties, and no interference was seen in the images. 

## 4. Capsule Endoscopy in Patients with Ventricular Assist Devices

Two case reports about patients with a left ventricular assist device (LVAD) who underwent CE are published in Medline. 

Girelli et al. report about 42-year-old patient with a history of an idiopathic dilatative cardiomyopathy, which was refractory to medical treatment [15]. An LVAD, Heart INCOR (Berlin Heart AG, Berlin, Germany), was implanted as a bridge to transplantation. Under a therapy with warfarin, aspirin, and low molecular weight heparin the patient suffered from melena. As upper and lower gastrointestinal endoscopy, splanchnic arteriography and tagged red blood cells scan were unrevealing. CE was performed. The parameters of LVAD were monitored during CE. No hemodynamic, electronic, or mechanical abnormalities or malfunction were observed. The video of CE was unaffected. 

Another case of a 45-year-old man with an LVAD (INCOR; Berlin Heart AG, Berlin, Germany) who suffered likewise from a dilatative cardiomyopathy was reported by Garatti et al. [16]. After implantation of the LVAD the patient developed melena. Numerous blood transfusions were necessary. Because upper and lower gastrointestinal endoscopy, catheterisation of superior and inferior mesenteric artery and tagged red blood cells scan were unable to detect a bleeding source. CE was performed. CE failed to reveal actively bleeding lesions. However, gastrointestinal bleeding did not recur after cardiac transplantation. The authors did not find any interference between CE and the Incor LVAD. 

## 5. Discussion

### 5.1. Capsule Endoscopy and Cardiac Pacemakers

Interference between CE and PM was investigated in 8 studies. In our second in vitro study [2], we simulated the situation of a pacemaker inside a patient, analogously to a study of Irnich et al. investigating the interference behaviour of mobile phones with respect to PM [3]. This setting is equivalent to a patient in contrast to the setting in our first in vitro study [4] and perhaps the study of Payeras with missing description of the concentration of saline solution [5]. Although it is problematic that the TestCap in the studies of Dubner and Payeras was located not in direct contact with the skin surface/into the saline solution causing that the setting is not equal to the circumstances inside a patient.

The study of Guyomar reports about a patient with an abdominal pacemaker programmed to VOO-Mode before swallowing CE [8]. This setting is problematic because the VOO-Mode is an interference mode. Interference between telemetry and CE could possibly explain loss of images in this study. Disturbance of a CE video caused by simultaneous telemetry was registered in an own multicenter survey [17]. Interference might be explained by the fact that many wireless applications like some telemetry use the same frequency as CE. 

Most studies preferred monitoring for the whole time of CE. In Dubner's study, interference occured within the first 10 seconds [7]. From a physical point of view it is clear that interference occurs at once or not. Because of this fact, long-term monitoring is not necessary. 

It has been argued by Payeras et al. [5] that a bipolar mode minimizes the risk of interference between CE and PM and that bipolar pacemakers would be resistant to interference from mobile phones (frequency: CE Given Imaging 434.09 MHz, CE Olympus 433.8 MHz, C-Net mobile phone 450 MHz) [18]. Irnich et al. investigated 44 Medtronic models in bipolar and unipolar mode and demonstrated that 5 out of 27 unipolar systems (18,5%) and 4 out of 17 (23,5%) bipolar systems were susceptible [3]. In another study, interference was only seen in patients with bipolar pacemakers [7]. The reason for interference (no interference in our study) between the “Actros” (Biotronik, Berlin, Germany) in the study by Dubner remains unclear, and the results seem to be physically rather dubious. 

Relating to possible interference between CE and PM/ICD we contacted the manufacturer Given Imaging. Technical data of CE were made available to the first author of this paper and Prof. Dr. Silny (Head of the Forschungszentrum für Elektro-Magnetische Umweltverträglichkeit, RWTH Aachen) after signature of a nondisclosure agreement. On the basis of these data Prof. Dr. Silny concluded that an interference between CE (Given Imaging) and PM/ICD is impossible from technical site even CE and PM/ICD are in close proximity (written statement of Prof. Dr. Silny). 

Two other types of capsule endoscopes are on the market. The OMOM capsule (Jinshan, Chongqing, China) has a similar transmission of images by radio frequency [19]. However, no data are available yet on the safety of this system in patients with pacemaker/ICDs. In contrast, the MiRo Cam (Intromedic, Seoul, Korea) is based on a totally different system of electric field propagation [20]. Images are transferred via 3 V current by using the human body as a conductor together with standard ECG electrodes attached to the abdomen. For this system, systematic studies are required before application in patients with pacemaker/ICDs.

Further developments of wireless capsule endoscopy systems include remote control of capsule functions via the recorder. The OMOM small bowel system enables manual switching between image acquisition rates, for example, for a power saving mode during gastric passage [21]. PillCam Colon2 provides automatic frame rate control (4 versus 39 images/sec.) depending on the speed of the capsule as represented by the changes of images [22]. Although interference with implanted cardiac devices is as unlikely as with signal sent from the capsule, no data has been published yet on in vitro or in vivo evaluation. 

A new capsule endoscopy system, presently under clinical evaluation (Capsovision, Saratoga, CA, USA) stores acquired image data on an internal chip. In consequence, the capsule has to be retrieved. On the other hand, by completely avoiding emission of current or radiofrequency waves, there is no possibility for potential interaction with implanted cardiac devices. 

### 5.2. Capsule Endoscopy and Implantable Cardioverter Defibrillators

Our investigation for interference between CE and ICD is the only one testing interference behaviour of ICDs [10]. As described above, the setting was chosen to electrically simulate the situation of a pacemaker inside a patient. The study of Dubner et al. used a “saline gel bath” without specification of the concentration of the saline solution. The TestCap and the saline solution/skin of the patient were not in direct contact in this study too [11]. One ICD, the “Belos” (Biotronik, Berlin, Germany), showed interference with CE (TestCap located up to 30 cm from the ICD) when the TestCap CE was placed over the ring and coil but not over the generator. This observation is inconsistent with a previous study of Dubner with interference between CE and PM if CE was located in close proximity (<10 cm) to the pacemaker generator [7]. In our study, no interference was noticed between CE and the “Belos” [10]. 

Based on the technical data of CE and impossibility of interference between CE and PM/ICD, the reported interference must be related to other (local) reasons. 

### 5.3. Capsule Endoscopy and Left Ventricular Assist Devices

Two case reports not report about interference between CE and LVAD. A multicenter US series investigation from the Mayo Clinic Scottsdale revealed interference between CE and LVAD in 2 patients [23]. The reason for this interference is not commented on by the authors. Perhaps the patients were monitored with telemetry. 

## 6. Conclusion

1. There are unconfirmed reports on possible interference of an electromagnetic device for simulation of capsule endoscope transmission (Given Imaging) on cardiac pacemakers and implantable cardioverter defibrillators in a setting of maximal susceptibility. However, other in vitro studies, theoretical considerations, and an increasing number of clinical observations support that there is no risk for patients with cardiac devices undergoing CE with the Given small bowel system. Preliminary data suggest that this might also be applicable for the Olympus system and for patients with left ventricular assist devices.
2. Further studies on OMOM capsule, MiRo Cam, and the remote controllable PillCam Colon2 are needed.
3. Interference between telemetry and impairment of CE video by wireless telemetry and left ventricular assist devices is possible.

## Figures and Tables

**Table 1:** Studies investigating patients with cardiac pacemakers who underwent capsule endoscopy.

Author	Year	Number of patients/cardiac pacemakers (*n*)	Brand of cardiac pacemaker	Kind of study	Interference	Brand of CE
Dirks et al.	2008	5	No specification	In vivo	No	Given Imaging
Bandorski et al.	2008	21	Medtronic, Osypka, Siemens, Vitatron, Ela, Guidant, St. Jude Medical	In vitro	No	Given Imaging + Olympus
Bandorski et al.	2006	1	Biotronik	In vitro	No	Given Imaging
Payeras et al.	2005	20	No specification	In vitro + In vivo	No	Given Imaging + Test Cap
Bandorski et al.	2005	45	No specification	In vivo	No	Given Imaging
Dubner et al.	2005	100	St. Jude Medical, Medtronic, Guidant, Biotronik, Sorin	In vivo	Yes (*n* = 4, noise mode)	Given Imaging
Guyomar et al.	2004	1	ELA	In vivo	No	Given Imaging
Leighton et al.	2004	5	No specification	In vivo	No	Given Imaging

**Table 2:** Studies investigating patients with implantable cardioverter defibrillators who underwent capsule endoscopy.

Author	Year	Number of patients/ICD (*n*)	Brand of ICD	Kind of study	Interference	Brand of CE
Bandorski et al.	2009	45	Biotronik, Guidant, Medtronic, St. Jude Medical	In vivo	No	Given Imaging + Olympus
Dubner et al.	2008	6 (In vitro) 6 (In vivo)	Medtronic, St. Jude Medical, Guidant, Biotronik	In vitro + In vivo	Yes No	Given Imaging
Pelargino et al.	2005	1	Medtronic	In vivo	No	Given Imaging
Bandorski et al.	2005	8	No specification	In vivo	No	Given Imaging
Leighton et al.	2005	5	Guidant, Medtronic, St. Jude Medical	In vivo	No	Given Imaging
